# Innovative Molecular Imprinting Sensor for Quick, Non-Invasive Cortisol Monitoring in Fish Welfare

**DOI:** 10.3390/bios15040204

**Published:** 2025-03-21

**Authors:** Hugo G. Santos, Daniela Santos Oliveira, Felismina T. C. Moreira

**Affiliations:** 1 CIIMAR—Interdisciplinary Centre for Marine and Environmental Research, University of Porto, 4460-314 Matosinhos, Portugal; hsantos@ciimar.up.pt; 2 CIETI-LabRISE, ISEP, Polytechnic of Porto, Rua Dr. António Bernardino de Almeida 431, 4249-015 Porto, Portugal; ddsol@isep.ipp.pt; 3 Facultade de Bioloxía, Universidade de Vigo, 36310 Vigo, Spain

**Keywords:** aquaculture, screen-printed carbon electrode (SPCE), European seabass, zebrafish, welfare assessment

## Abstract

The assessment of fish welfare is crucial to prevent economic losses in aquaculture and ensure reliable results in research. A quick, non-invasive device to measure cortisol levels in fish farm water facilitates welfare evaluation and corrective actions when compromised. To address this need, an innovative sensor was developed using screen-printed carbon electrodes (SPCEs) functionalized with reduced graphene oxide/Prussian blue nanocubes (rGO/PBNCs) for direct selective detection of cortisol. A molecularly imprinted polymer (MIP) was synthesized on rGO/PBNCs/SPCEs by electropolymerization (ELP) of pyrrole in the presence of cortisol. The polymerization solution was prepared by adding cortisol (5 mM) and pyrrole (0.3 M) to a DMF/PBS (1:4) solution (pH 7.4). Following ELP, the electrodes were washed with PBS, and pyrrole overoxidation was used to extract cortisol from the polymer matrix. For comparison purposes, a non-imprinted polymer (NIP) was also fabricated. The electrodes were characterized using scanning electron microscopy (SEM) and Raman spectroscopy to assess their morphological and chemical features. Under optimized conditions, the sensor showed a linear range from 0.1 nM to 0.1 mM in artificial saltwater. This sensor combines simplicity and affordability while providing reliable detection of chemical and biological compounds.

## 1. Introduction

Aquaculture and scientific research are two different fields where fish have gained increasing importance, namely in protein production [1,2] and human health discoveries [3,4]. The detection of cortisol, a primary stress biomarker in fish, in aquatic environments marks a significant advancement in fish welfare assessment [5]. The development of molecularly imprinted polymer (MIP) sensors for non-invasive measurement of cortisol in fish rearing water will be decisive in aquaculture productivity and fish health [6]. Cortisol provides critical insights into the physiological state of fish, but traditional methods for cortisol assessment involve invasive procedures (blood collection) that can stress the fish [7]. MIP technology may offer a promising non-invasive alternative, allowing for real-time monitoring of cortisol levels directly from the rearing water. This technology has also been developed for evaluating human stress through cortisol measurement in sweat [8,9]. MIPs are synthetic polymers constructed to possess selective recognition sites for specific molecules, where the polymer matrix forms around a target molecule (template), creating a cavity that is complementary in shape, size, and functional groups to the template molecule. Upon removal of the template, the polymer retains these cavities, enabling it to selectively rebind to the target molecule with high affinity [10]. These imprinting procedures on synthetic receptors give them a unique combination of properties such as robustness, high affinity, specificity, and low-cost production, which makes them interesting alternatives to natural receptors. Building MIPs in sensor platforms has been explored for various applications, including environmental monitoring and biomedical diagnostics [11,12].

Commonly, cortisol levels in fish are measured in blood by using enzyme immunoassay (EIA) or radioimmunoassay (RIA) techniques [13]. Wu H. et al. [14] developed an immunosensor for detecting fish plasma cortisol, which had the advantage of being a rapid and convenient method to analyze fish plasma samples. However, the use of this immunosensor still implied blood collection, an invasive procedure. Throughout the years, several authors have presented the utility of measuring cortisol in fish rearing water as a welfare assessment tool in several different species, such as common carp (*Cyprinus carpio*) [15], rainbow trout (*Oncorhynchus mykiss*) [16], Atlantic salmon (*Salmo salar*) [17], European seabass (*Dicentrarchus labrax*) [18], zebrafish (*Danio rerio*) [19], and Nile tilapia (*Oreochromis niloticus*) [20]. Cortisol levels in fish rearing water showed normal values between 0 and 10 ngL^−1^ for most of the species named previously. Nevertheless, whenever the fish were subjected to different stress stimuli (e.g., high stocking density, capture and release, exposure to air, etc.), the water cortisol levels increased significantly, namely up to 140 ng L^−1^ for European sea bass [18] or up to 275 ng L^−1^ for Asian sea bass [21]. Despite being a non-invasive way of assessing fish welfare, water cortisol measurement was still dependent on immunoassay techniques, which are time-consuming and do not allow real-time welfare monitoring.

The use of MIPs for measuring cortisol in rearing water has not been reported. Nevertheless, recent advancements in MIP sensors for cortisol detection for human health monitoring (through cortisol detection in sweat and saliva) [22,23] have demonstrated the potential for their application in monitoring fish welfare. Klangphukhiew S. et al. [24] developed a cortisol MIP using a multi-step swelling and polymerization process, which was integrated onto a disposable screen-printed carbon electrode for electrochemical analysis. This sensor presented high selectivity and sensitivity for cortisol, indicating that MIP-based sensors may be used for aquatic organism welfare assessment. Similarly, the production of a cortisol-specific MIP sensor should be supported with the optimization of some important parameters, such as the monomer concentration or the number of electropolymerization cycles. Using electrochemical techniques, such as differential pulse voltammetry, this MIP sensor achieved a limit of detection of 0.036 nM while confirming the specific binding affinity towards cortisol [25].

The application of MIP sensors for cortisol detection in both aquaculture and laboratory settings represents a transformative approach to fish welfare assessment. Our work presents a fish welfare molecular imprinting-based sensor that will be a non-invasive, sensitive, and selective solution for real-time cortisol monitoring in fish rearing water. Data obtained with this cortisol-specific sensor, together with other welfare indicators, can significantly enhance our understanding of stress dynamics in aquatic organisms, as well as allowing a faster and more efficient response to impaired welfare situations. Furthermore, as a non-invasive technique to monitor fish well-being, it will be aligned with ethical considerations and improve the overall management of aquaculture and laboratory systems. The new fish welfare sensor will be an important tool for both farmers and researchers, ensuring the production of high-quality fish food and the reliability of scientific data, respectively.

## 2. Materials and Methods

### 2.1. Apparatus and Electrodes

Electrochemical analyses were performed using a PalmSens4 potentiostat (Houten, The Netherlands). The screen-printed carbon electrodes (SPCEs), model DRP-110, were purchased from Metrohm DropSens ((Metrohm/DropSens220AT, Oviedo, Spain). These electrodes consisted of a 4 mm diameter carbon working electrode (WE), a carbon counter electrode (CE), and a pseudo-reference electrode (RE) made of silver.

For morphological characterization, scanning electron microscopy (SEM) was conducted with an FEI Quanta 400 FEG ESEM (Eindhoven, The Netherlands) under high vacuum conditions at 15 kV and a magnification of 200×. Images were obtained from multiple regions of each sample, including the unmodified SPCE, rGO/PBNCs, and both MIP and NIP materials.

Raman spectroscopy was performed on dried supernatant droplets using a Renishaw inVia microscope (Gloucestershire, UK) with a 532 nm excitation laser and a 100× objective, giving an approximate laser spot size of 1 μm^2^. The applied laser power was approximately 5 mW, and calibration was performed using the 520 cm^−1^ peak of a silicon wafer. The images were taken from different locations of each sample, particularly emphasizing the unmodified SPCE and the MIP and NIP materials.

### 2.2. Reagents and Solutions

All reagents were of analytical grade, and ultrapure deionized (DI) water was used in all experiments. Cortisol was purchased from Merck (Darmstadt, Germany), while potassium chloride (KCl) was supplied by Normapur (Leuven, Belgium). Potassium hexacyanoferrate (III) (K_3_Fe(CN)_6_) and potassium hexacyanoferrate (II) trihydrate (K_4_Fe(CN)_6_•3H_2_O) were obtained from Riedel-de-Haën (Seelze, Germany). Phosphate-buffered saline (PBS) in tablet form (10 mM, pH 7.4) was obtained from VWR. Cholesterol was purchased from ThermoFisher (Loughborough, UK). Sulfuric acid (98%), poly (ethyleneimine) (PEI), graphite, 17α-methyltestosterone, and dimethylformamide (DMF) were purchased from Sigma Aldrich (Gillingham, UK). Pyrrole (99%) and bovine serum albumin (BSA) were purchased from Alfa Aesar (Heysham, England). Oxytetracycline (Oxykel 80%) was obtained from MVAqua (Aveiro, Portugal). Cortisol and cholesterol are poorly soluble in aqueous buffers. To maximize solubility in aqueous buffers, cortisol and cholesterol were first dissolved in DMF and then diluted with PBS in a 1:4 solution of DMF/PBS. The Tropic Marin Premium REEF salt (Wartenberg, Germany) purchased was selected for this study because its composition closely resembles the chemical composition of natural seawater, including minor ions (e.g., bromide, fluoride, and bicarbonate) and trace elements (e.g., iron, copper, and zinc), ensuring a very accurate and reliable simulation of marine conditions.

### 2.3. Graphene Oxide/Prussian Blue Nanocube (GO/PBNC) Nanomaterial Synthesis

Biographene was synthesized following the procedure outlined by Erdőssy et al. [26]. In brief, a suspension of graphite crystals (100 mg mL^−1^) was prepared in 200 mL of DI water at pH 7.0, containing 3.0 mg mL^−1^ of protein. This mixture was then processed in a kitchen blender for 30 min. To assess the exfoliation rate, aliquots were collected every 5 min, and the blender was paused intermittently to prevent overheating, ensuring the temperature remained below 30 °C. The graphene concentration was determined by measuring the absorbance at 660 nm after centrifugation at 1500 rpm for 45 min to remove non-exfoliated graphite. All exfoliation trials were conducted by precisely adding the calculated amounts of graphite, BSA, and DI water into the blender, with the blade speed set accordingly.

The nanocomposite of GO with PBNCs was synthesized using a straightforward method adapted from Yuqing et al. [27]. Under continuous stirring, 10 mL of FeCl_3_ (5 mM, pH 1.1), 1 mL of PEI (3%), and 10 mL of K_3_Fe(CN)_6_ (5 mM, pH 1.1) were sequentially added to a 10 mL dispersion of biographene (0.5 mg mL^−1^, pH 1.1). The resulting mixture was heated and kept under reflux for 3 h, and a color change from yellow to dark blue was observed, confirming the successful formation of the nanocomposite.

To purify the product, the mixture was centrifuged three times with ultrapure water. Finally, a clean SPCE was modified by dropping 3 μL of the synthesized rGO/PBNC nanocomposite onto the working electrode. The electrode was then dried for 20 min at 60 °C in a muffle furnace (Nabertherm, Lilienthal, Germany), and this process was repeated three times to ensure uniform coating.

### 2.4. Construction of Molecularly Imprinted Polymers (MIPs) for Cortisol

First, the electrodes were washed with ultrapure water. To improve the adhesion of the nanomaterial, the carbon electrode was pretreated with chronoamperometry (CA) in 0.1 M KCl for 400 s at +1.7 V. The nanocomposite was then deposited on the working electrode as described in Section 2.3. The electrodes were washed with ultrapure water and dried at room temperature. The MIP films were prepared by electropolymerization (ELP) using cyclic voltammetry (CV), where a potential range of −0.2 to +0.9 V was applied for three cycles at a scan rate of 50 mV s^−1^. The polymerization solution consisted of 5 mM cortisol and 0.3 mM pyrrole dissolved in buffer (DMF/PBS (1:4) mixture at pH 7.4).

**Figure 1:**
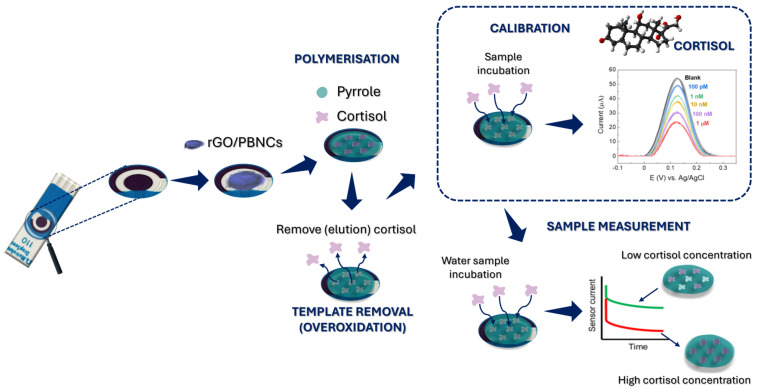
Schematic representation of the different steps necessary for the cortisol MIP sensor fabrication, calibration, and sample measurement.

After electropolymerization, the electrodes were rinsed with PBS, and pyrrole overoxidation was performed to remove the cortisol from the polymer matrix (Figure 1). This process was carried out using CV at a potential window of −1.0 to +1.0 V in 10 mM PBS for 10 cycles at a scan rate of 50 mV s^−1^. The electrodes were then thoroughly rinsed with PBS and air-dried at room temperature. As a control, films of non-imprinting polymers (NIPs) were prepared under the same conditions, but without the addition of cortisol.

### 2.5. Sensor Electrochemical Characterization and Performance Evaluation

The electrochemical characterization of the sensor design was carried out using CV. For the electrochemical measurements, 70 μL of a solution containing PBS (pH 7.4) and 5 mM [Fe(CN)6]^3−/4−^ was added as a redox probe. Calibration curves were generated by incubating the working electrode with 5 μL of a cortisol solution at different concentrations for 10 min. The solutions were prepared by adding 10% of the total volume of artificial saltwater to a PBS/DMF (4:1) solution. A 1 mM solution was prepared by adding 8 µL of a 25 mM cortisol solution to 192 µL of the previous solution. Subsequent concentrations were prepared by consecutively mixing 20 µL of the previous solution with 180 µL of the PBS/DMF and artificial saltwater solution. In this way, using the 1 mM concentration solution as the basis, the following cortisol concentration solutions were prepared: 100 µM, 10 µM, 1 µM, 100 nM, 1 nM, and 100 pM. Subsequent measurements were performed using square wave voltammetry (SWV) at room temperature at a potential range of −0.1 to +0.3 V. The SWV conditions included a pulse amplitude of +0.1 V, a pulse width of 50 ms, and a scan rate of 10 mV s^−1^. To ensure complete coverage of all three electrodes, 70 μL of a PBS/KCl solution was added before performing the SWV measurements for direct analysis, and 70 μL of a solution containing PBS/KCl (pH 7.4) and 5 mM [Fe(CN)6]^3−/4−^ was added for indirect readings.

### 2.6. Selectivity Studies

The selectivity percentage was determined by comparing the electrochemical signal of the cortisol solution alone with that of the cortisol solution mixed with an interference agent. To evaluate the selectivity of the developed sensors, their SWV responses to a 1 mM cortisol solution were analyzed in both the presence and absence of interferents using the competitive method. For this analysis, 1 mM cortisol solutions were prepared with the addition of cholesterol (0.1 mM), testosterone (0.1 mM), and oxytetracycline (0.1 mM). The SWV technique was then performed for each solution according to the previously described procedure. All solutions were prepared in synthetic aquaculture water diluted 10-fold in buffer.

Considering that the electrochemical signal of the 1 mM cortisol solution accounts for 100% of the reaction, the interference effect was calculated using Equation (1):

(1) $$\%interference=\left(\frac{Signal\left(cortisol\right)\;-\;signal\left(cortisol+interferent\right)}{Signal(cortisol)}\right)\times 100$$

## 3. Results and Discussion

Cortisol sensors were developed by immobilizing a layer of electroactive nanocomposite rGO/PBNCs onto the working electrode of SPCEs. The integration of rGO/PBNCs eliminates the need for a liquid redox solution, simplifying the sample analysis process. These rGO/PBNCs serve as an intrinsic redox mediator, simplifying the biosensor by removing the necessity for liquid redox probes. This innovative approach enables direct sample analysis, reducing the complexity of the device and making it more efficient and user-friendly.

The MIP-based cortisol sensor was produced by modifying the working electrode with the electroactive rGO/PBNC nanomaterial. After that, pyrrole was electropolymerized in the presence of cortisol molecules to create an MIP layer on the modified electrode. The removal of cortisol from the MIP film was accomplished by overoxidation in PBS, creating binding sites. Finally, cortisol was used to fill the preformed binding sites, allowing it to rebind (Figure 1).

### 3.1. Electrochemical Follow-Up of MIP Assembling

**Figure 2:**
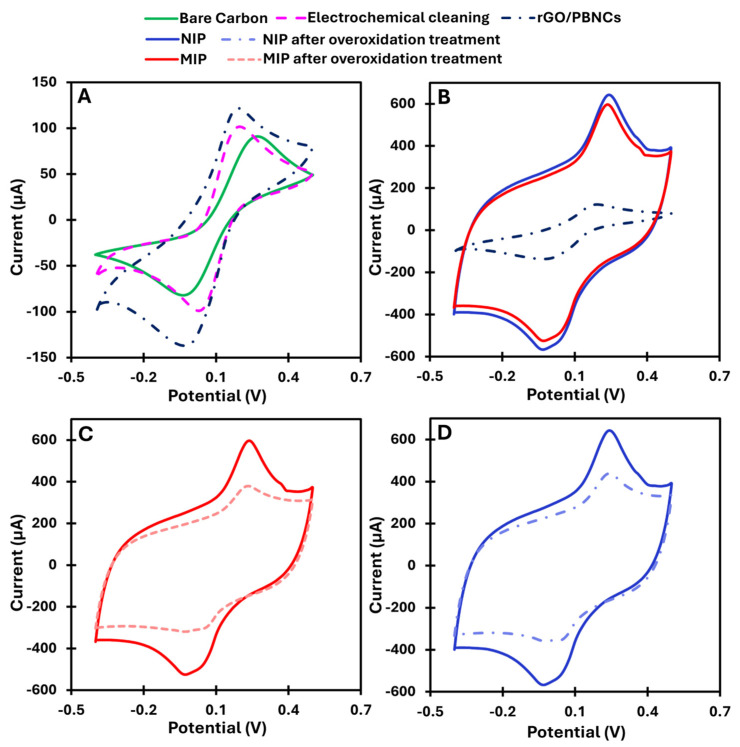
CV measurements were performed at various stages of the fabrication process for the MIP and NIP. (**A**,**B**) The CV response following the immobilization of the rGO/PBNC nanocomposites and subsequent formation of the MIP and its non-imprinted control. (**C**) The CV response of the MIP after the removal of the target molecule, and (**D**) the corresponding response for the NIP.

Drop-casting the synthesized nanomaterials onto the working electrodes provided a conductive and electroactive surface due to the presence of rGO/PBNC nanocomposites. After immobilization, an increase in the peak current and a decrease in the peak-to-peak separation in the CV were observed (Figure 2A). This indicates that the nanomaterials could favor electron transfer and consequently improve the sensitivity of the sensor.

MIP synthesis was performed by ELP of pyrrole mixed with cortisol. ELP was performed by CV scans between −0.1 and +0.9 V at a scan rate of 50 mV s^−1^. For comparison, NIP was prepared by following the same protocol but without using the target molecule. The ELP of MIP and NIP led to the formation of a highly conductive polymer, which was not expected at neutral pH. A possible explanation could be that the rGO/PBNCs, due to their redox properties, facilitate the charge transfer between the electrode and the polymer, thus improving the efficiency of the polymerization. Pyrrole forms a conjugated structure that conducts electrons during polymerization, while the interaction with rGO/PBNCs creates a redox network that increases the electrical conductivity of the polymer. The rGO/PBNC modification and the neutral pH value thus promote the formation of a highly conductive polymer. This combination provides excellent electrochemical performance at neutral pH, making it ideal for sensors operating under physiological conditions.

According to Figure 2B, the MIP shows a slight difference in current compared to the NIP, which is due to the low molecular weight of cortisol. Since cortisol is a small molecule, it is expected that there is no significant difference in the total net current.

After the removal of cortisol from the polymer matrix, a decrease in net current was observed in the CV spectra (Figure 2C,D). Normally, removal of the template from the polymer should lead to an increase in net current, not a decrease. This unexpected result can be explained by the chemical changes in the polypyrrole (PPy) caused by the overoxidation process during template removal. During this process, hydroxyl (-OH), carbonyl (C=O), and carboxyl (-COOH) groups are formed at the β-positions of the pyrrole ring when exposed to high potentials in the CV range of −0.1 to +1.0 V in PBS [28]. In addition, the formation of non-conducting groups, such as carbonyl groups, and changes in the redox state of the polymer reduce its ability to conduct electricity, resulting in a loss of conductivity. Overoxidation also creates cavities in the polymer that are complementary to cortisol, facilitating its subsequent detection [28].

### 3.2. Chemical and Morphological Surface Characterizations

#### 3.2.1. Raman Spectroscopy

**Figure 3:**
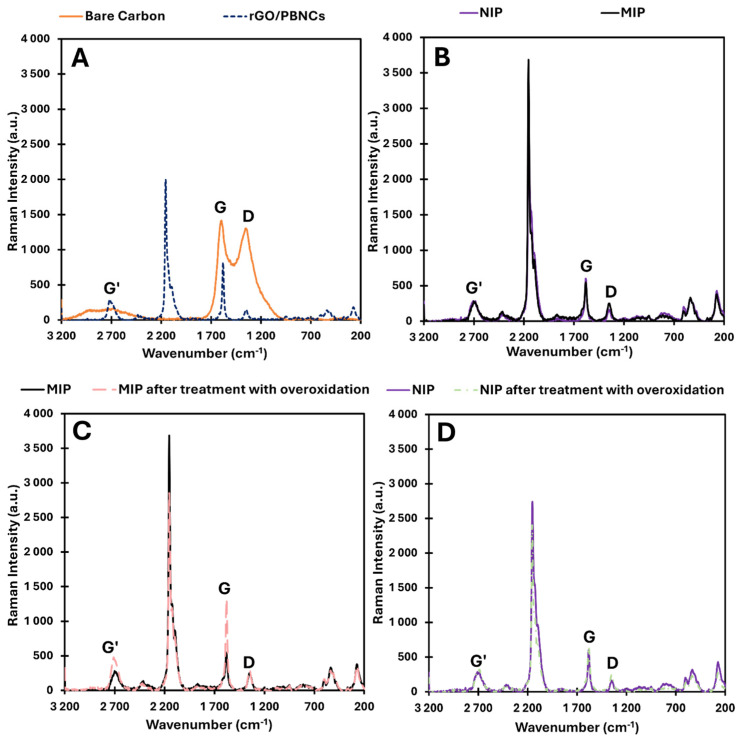
Raman spectroscopy for (**A**) SPCE and SPCE/rGO/PBNCs, (**B**) SPCE/rGO/PBNCs/MIP, SPCE/rGO/PBNCs/NIP, (**C**) SPCE/rGO/PBNCs/MIP and SPCE/rGO/PBNC/MIP after treatment with overoxidation, and (**D**) SPCE/rGO/PBNC/NIP and SPCE/rGO/PBNC/NIP after treatment with overoxidation.

Figure 3 shows the Raman spectra for SPCE/rGO/PBNCs, SPCE/rGO/PBNCs/MIP and SPCE/rGO/PBNCs/NIP, MIP, and NIP after removal of the template (overoxidation process).

The analysis of the bare electrode showed the presence of the G peak at 1580 cm^−1^ and the D band at 1340 cm^−1^, which indicates the carbon matrix presence. After casting the nanocomposite on the bare electrode surface, the presence of Prussian blue was observed, showing characteristic peaks between 2070 and 2160 cm^−1^ corresponding to C≡N triple bond vibrations. These characteristic peaks were also observed in the MIP and NIP sensors, as they were built on SPCEs modified with rGO/PBNCs [29,30,31]. Raman spectroscopy is an essential method for analyzing PBNCs, especially by detecting the ν(CN) bands within the 2157–2160 cm^−1^ range [32]. These bands offer valuable information about the degradation of Prussian blue, as the vibrational stretching mode’s wavenumber varies depending on the iron oxidation state. Therefore, examining the ν(CN) peaks in the Raman spectrum allows for the evaluation of PB’s redox state. In this spectral region, the C≡N group, which is coordinated with iron ions in different valence states, shows a variety of ν(CN) Raman vibrations. The primary peak, located between 2157 and 2158 cm^−1^, corresponds to the 1A g ν(CN) stretching vibration and the [Fe(II), Fe(III)] state. This peak shows a shoulder at lower wavenumbers (around 2125 cm^−1^), which is characteristic of the CN group.

The I_D_ and I_G_ bands were present on the surface of all electrodes with rGO/PBNCs, except for the bare carbon electrode. These bands are very important and provide crucial information about the structural features and electrochemical properties of carbon electrodes. The I_D_/I_G_ ratio is an important indicator of the structural quality and electrochemical properties of carbon-based materials. A higher ratio usually means more defects and more surface reactivity, while a lower ratio indicates better conductivity and a more ordered carbon structure. The I_D_/I_G_ ratios for each material were (i) 0.92 for bare carbon, (ii) 0.18 for SPCE/rGO/PBNCs, (iii) 0.46 for SPCE/rGO/PBNCs/MIP, (iv) 0.27 for SPCE/rGO/PBNCs/NIP, (v) 0.21 for MIP after treatment with overoxidation, and (vi) 0.38 for NIP after treatment with overoxidation.

When we used the rGO/PBNC nanocomposite, the defects decreased to 0.18. The decrease in defects after immobilization of the nanocomposite of rGO/PBNCs on the carbon electrode is likely due to a combination of surface passivation, structural reorganization, and enhanced electron transfer properties imparted by the nanocomposite. The rGO/PBNCs and graphene help to stabilize and repair the carbon surface, reducing disorder and resulting in a more ordered and less defective material, which is reflected in the Raman spectrum with a lower I_D_/I_G_ ratio. After MIP and NIP assembly, an increase in defects was observed, which could be primarily due to ELP-induced stresses, interactions between the polymer and the underlying nanocomposite, and the formation of new functional groups or changes in electronic structure during the polymerization process. These factors contribute to a more disordered surface, which is reflected in an increased D-band intensity and a higher I_D_/I_G_ ratio in Raman spectroscopy. While the previous modifications (rGO/PBNCs) aimed to improve the structural order, the electropolymerization of PPy introduced new complexities, leading to a higher defect density. After matrix removal, the MIP and NIP polymers were oxidized, and a decrease in structural defects in the polymer matrix was observed, consistent with the absence of cortisol in the polymer.

#### 3.2.2. SEM Analysis

**Figure 4:**
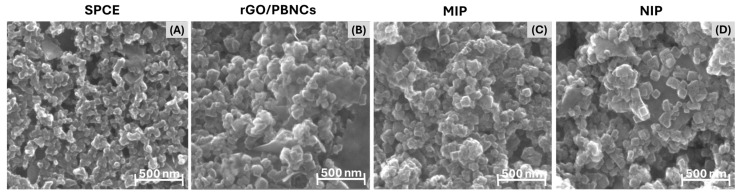
SEM images for (**A**) SPCE, (**B**) SPCE/rGO/PBNCs, (**C**) SPCE/rGO/PBNCs/MIP and (**D**) SPCE/rGO/PBNCs/NIP materials.

Morphological analysis of the SPCEs, rGO/PBNCs, and MIP and NIP materials was performed using SEM, and the results are shown in Figure 4. The SEM images clearly show the formation of nanocubes with an average length between 30 and 100 nm. Furthermore, the presence of graphene sheets within the nanocomposite material can be observed, indicating the successful integration of graphene oxide (GO) into the nanocomposite structure.

Graphene sheets are clearly visible in the nanocomposite material, showing the successful integration of graphene oxide (GO) into the nanocomposite structure (Figure 4B). However, the MIP layer polymerized on the surface of the rGO/PBNCs is not clearly visible (Figure 4C). This can be attributed to two factors. First, the inherent non-uniformity of the rGO/PBNCs results in a surface with different topographies and sizes, making it difficult to uniformly coat the surface with the polymer. Secondly, the PPy film formed during ELP is likely to be very thin, making it difficult to visualize by SEM. The combination of the thickness of the PPy layer, the porosity, and the possible molecular interactions with the rGO/PBNCs results in a structure where the polymer layer merges with the underlying nanocubes, making them even more difficult to distinguish.

### 3.3. Analytical Performances of the Biosensors

#### 3.3.1. Calibration in Buffer

When cortisol molecules rebind to the imprinted PPy, the cavities are filled, blocking the redox probe’s access to the electrode surface and inhibiting electron transfer, resulting in a reduced electrochemical signal. SWV was used to measure the cortisol concentration in the buffer as it effectively minimizes electrochemical interference and increases the sensitivity of the sensor by eliminating the charging current. The calibration curve was plotted by relating the cortisol concentration to the corresponding change in peak current of the SWV curves (ΔI/I_0_) and serves as a reference for evaluating the performance of the MIP sensor. A steeper calibration curve indicates higher sensitivity of the sensor for cortisol.

**Figure 5:**
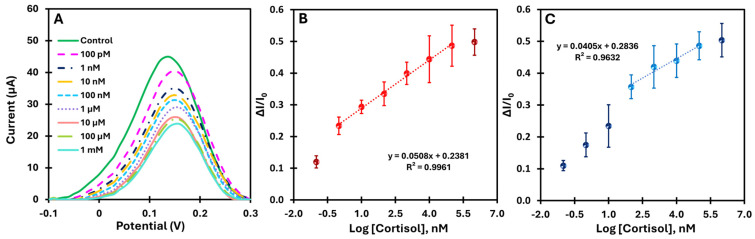
Electrochemical characterization of the MIP-based biosensor using SWV (**A**), along with the corresponding calibration curve (**B**) and the corresponding calibration curve for the NIP sensor (**C**), obtained by incrementally increasing the cortisol concentration in buffer.

Both the MIP and NIP sensors were tested for analytical performance by incubating 5 µL of cortisol solutions with increasing concentrations on the working electrode for 10 min. Figure 5A shows the change in current with increasing cortisol concentration, demonstrating the sensitivity of the sensor over a wide range. The calibration curves (Figure 5B) show the current as a function of the logarithm of the cortisol concentration for MIP over the range from 100 pM to 1 mM at pH 7.4.

The MIP-based platform showed a consistent decrease in current upon rebinding of the target, with a well-defined linear response across a broad concentration range (1 nM to 100 µM). The high correlation coefficient (R^2^ > 0.99) indicates excellent linearity. In addition, the limit of detection (LoD) was determined by finding the point where the two linear segments of the electrical response versus concentration curve intersect [33].

The LoD obtained was approximately 3.16 pM, confirming the platform’s high sensitivity. In comparison, the NIP-based platform (Figure 5C) showed a correlation coefficient of R^2^ = 0.96 within the same concentration range (100 nM to 100 µM), indicating lower linearity. These results highlight the significant improvement in the electrical response of the MIP platform due to the molecular imprinting of cortisol and demonstrate the superior analytical performance compared to the control.

#### 3.3.2. Calibration in Artificial Saltwater (Tropic Marin Salt)

**Figure 6:**
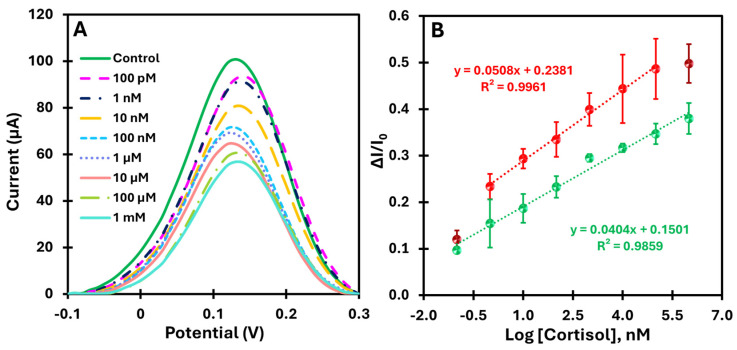
Electrochemical characterization of the MIP-based biosensor using SWV (**A**) along with the corresponding calibration curve (**B**), obtained by incrementally increasing the cortisol concentration in PBS (red dots) and artificial saltwater (green dots).

After calibrating the biomimetic sensor in a buffer, we moved on to testing it in a more realistic environment. For this purpose, the calibration curve was determined in an artificial saltwater environment enriched with cortisol in the range of 100 pM to 1 mM. This environment simulates practical conditions, allowing the robustness and sensitivity of the sensor to be evaluated in a more complex matrix. Figure 6A shows the current variations as a function of increasing cortisol concentration in the artificial saltwater samples and illustrates the sensitivity of the sensor over a wide range of concentrations. The calibration curves in Figure 6B show the current as a function of the logarithm of the cortisol concentration in two different matrices: PBS (red dots) and artificial saltwater (green dots). Both conditions were analyzed in the range of 100 pM to 1 mM.

The MIP-based platform showed a well-defined linear response with a correlation coefficient above 0.98 for both matrices, indicating good linearity and reproducibility (error bars less than 10%). The detection limit in the more complex matrix was 10 times lower than that in the buffer assay, reflecting a higher sensitivity in more complex media. In comparison, the analysis in artificial saltwater showed a slightly lower slope compared to the buffer, indicating a slight decrease in sensitivity due to the presence of potential interferents in the more complex matrix. Thus, these results confirm that the MIP-based platform provides better analytical performance with greater selectivity and electrical response compared to the control (NIP).

#### 3.3.3. Proof of Concept—Direct Method

**Figure 7:**
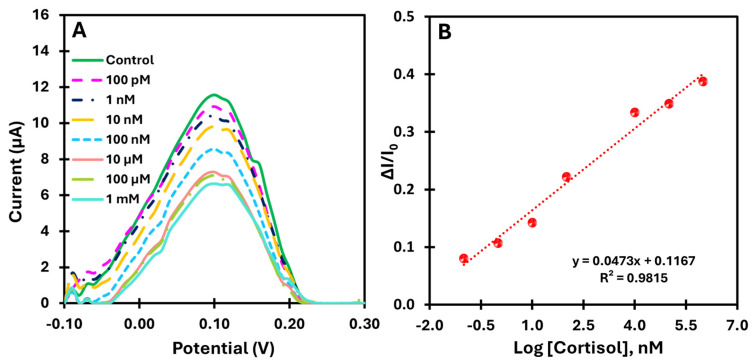
Electrochemical characterization of the MIP-based biosensor using SWV (**A**), with measurements performed for different cortisol concentrations in buffer without the use of a redox probe. (**B**) The corresponding calibration curve obtained for MIP.

For the direct measurement of cortisol using the catalytic properties of the rGO/PBNCs compound, the biomimetic sensor was evaluated by directly analyzing cortisol prepared in buffer. This approach was used to demonstrate the feasibility of real-time monitoring of cortisol without external redox probes. The calibration of the sensor was performed with cortisol solutions in a wide concentration range from 100 pM to 1 mM. This allowed the evaluation of the sensitivity and dynamic response of the sensor under conditions relevant to practical applications. Figure 7A shows the SWV voltammograms for the different cortisol concentrations tested. A consistent decrease in peak current was observed with increasing cortisol concentration, confirming the sensor’s ability to detect cortisol directly and continuously. Figure 7B shows the corresponding calibration curve plotting the relative change in current (ΔI/I_0_) against the logarithm of cortisol concentration. The sensor showed a linear response in the range of 100 pM to 1 mM, with a slope of 0.0473 log[cortisol] and a lower limit of detection of 23.0 pM.

Overall, as the concentration of cortisol increases, more cavities are filled in the MIP, changing the porous structure of the material and hindering the diffusion of the electrolyte to the electrode surface. A possible explanation for this mechanism could be that ion diffusion in the electrolyte is impeded when the protein binds to the MIP, leading to a decrease in ion mobility near the electrode surface. Several factors could contribute to this phenomenon. First, the binding of the protein may physically block the electrode surface and prevent the ions from reaching the electrode efficiently. This presents an obstacle to the electrochemical reaction. In addition, the binding could alter the structure of the double layer and the gradient of ion concentration near the electrode, making the normal charge transfer process more difficult. Finally, the presence of the protein increases the local resistance to ion flow, which further hinders the movement of ions at the electrode–electrolyte interface. As a result of these changes, the electrochemical current decreases, reflecting the reduced availability of ions for the redox reaction.

The results obtained underline the promising analytical feature of the proposed method for direct monitoring of cortisol levels. By exploiting the intrinsic catalytic properties of the rGO/PBNCs nanocomposite, the method eliminates the need for external redox probes, simplifying the analysis while ensuring high sensitivity and accuracy.

Several published studies have investigated MIP-based sensors for the detection of cortisol by electrochemical transduction, demonstrating remarkable advantages (Table 1). However, most of these studies focused on biomedical applications and involved more complex fabrication and detection techniques. In contrast, the main strength of our work lies in its simplicity, cost-effectiveness, and direct applicability in aquaculture. Our sensor provides a practical solution for monitoring cortisol in fish farm water, enabling real-time assessment of animal welfare with minimal operational complexity. This distinguishes it from other MIP-based cortisol sensors, which are mostly developed for biomedical purposes and require more elaborate methods.

### 3.4. Selectivity Study

**Figure 8:**
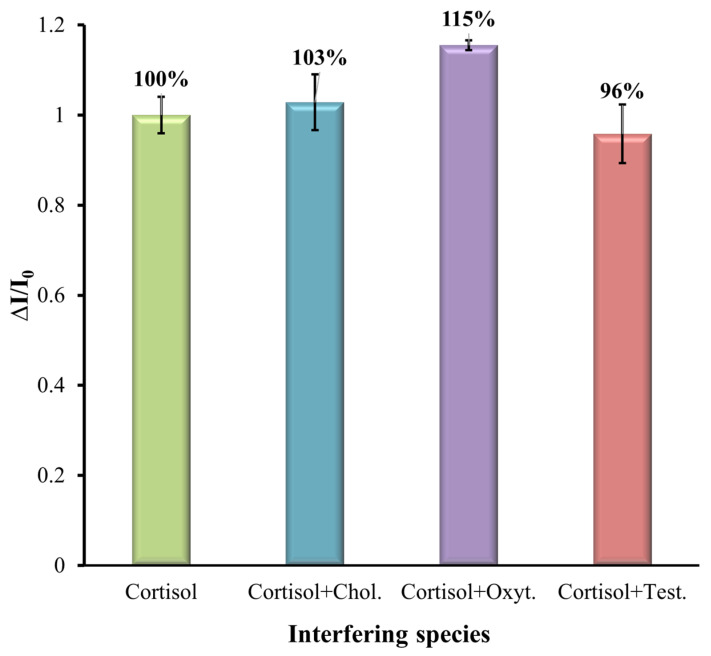
Relative current intensity data extracted from the SWV plots for cortisol standard solutions and other possible interfering species.

Here, the selectivity of the MIP-based sensor was analyzed for some common interferences such as cholesterol (Chol.), oxytetracycline (Oxyt.), and testosterone (Test.), as these may coexist in aquaculture water. The SWV analysis was performed to verify the selectivity of the sensor. To mimic real conditions, the interference elements were prepared with a mixed solution. The cortisol solution with a concentration of 1 mM was mixed with different interferent species at a concentration of 0.1 mM and prepared in artificial saltwater diluted by 10 with a pH of 7.4 and analyzed with the modified MIP SPE. The results are shown in Figure 8. The results show that even in the presence of interfering compounds together with cortisol, they had little effect on the determination of cortisol together with the MIP-modified electrode. The results show that the % of interference in the MIP-modified electrode for cholesterol, oxytetracycline, and testosterone was 3%, 15%, and 4%, respectively, indicating the presence of template-shaped cavities on the MIP. This selectivity parameter is good for the clinical applicability of the prepared MIP sensor.

## 4. Conclusions

This study introduced an innovative biomimetic sensor designed for the direct, continuous, and cost-effective measurement of cortisol levels in aquaculture environments, addressing a critical need for real-time fish welfare monitoring. The sensor utilizes a screen-printed carbon electrode (SPCE) functionalized with graphene oxide/Prussian blue nanocubes (rGO/PBNCs), a nanocomposite material that endows the system with exceptional catalytic properties, enabling high sensitivity and selectivity in cortisol detection. A key advancement of this sensor is the incorporation of an MIP layer on the electrode surface, synthesized via electropolymerization of pyrrole in the presence of cortisol molecules. This MIP layer ensures the highly selective recognition of cortisol, providing accurate and reliable measurements even in complex artificial saltwater samples. Under optimized conditions, the sensor demonstrated an impressive linear detection range (0.1 nM to 0.1 mM), enabling the direct and continuous monitoring of cortisol without the need for external reagents or probes. The device’s excellent performance highlights its ability to adapt to real-world applications, particularly in aquaculture settings where rapid and non-invasive stress monitoring is crucial.

The combination of the rGO/PBNCs nanocomposite material and the MIP layer ensures a robust and efficient sensing platform, offering simplicity, rapid response, and low production costs. This makes the sensor a transformative tool for continuous cortisol monitoring, not only in aquaculture environments but also in other scenarios where cortisol serves as a biomarker for stress or health conditions. By providing a strategic and versatile platform for the detection of cortisol, this sensor has significant implications for improving fish welfare, optimizing aquaculture management practices, and advancing scientific research. The direct and continuous monitoring capabilities offered by this approach represent a major step forward in sustainable aquaculture and biomarker detection technologies. Future studies and real-world applications will further validate the sensor’s potential for widespread adoption across industry and research sectors.

## Figures and Tables

**Table 1:** Analytical performances of MIP-based sensors reported in the literature for the detection of cortisol (2020–2024).

Sensing Approach	Transducer	Response Range	LOD (pM)	Detection	Reference
MIP Sensor	Electrochemical	0.5–64 nM	140	Indirect	[34]
MIP Sensor	Electrochemical	1 pM–10 nM	0.33	Indirect	[35]
MIP Sensor	Electrochemical	0.05 nM–2.5 µM	16	Indirect	[36]
MIP Sensor	Electrochemical	10^−12^–10^−8^ M	0.314	Indirect	[37]
MIP Sensor	Electrochemical	0.417 nM–1.28 μM	417	Direct	[38]
MIP Sensor	Electrochemical	10–450 nM	1070	Direct	[39]
MIP Sensor	Electrochemical	0.1 pM–1 μM	0.1	Direct	[40]
**MIP Sensor**	**Electrochemical**	**0.1 nM–0.1 mM**	**23**	**Direct**	**This Work**

## Data Availability

Data are contained within the article.
